# Deacetyl Ganoderic Acid F Inhibits LPS-Induced Neural Inflammation via NF-κB Pathway Both In Vitro and In Vivo

**DOI:** 10.3390/nu12010085

**Published:** 2019-12-27

**Authors:** Feiya Sheng, Lele Zhang, Songsong Wang, Lele Yang, Peng Li

**Affiliations:** 1 State Key Laboratory of Quality Research in Chinese Medicine, Institute of Chinese Medical Sciences, University of Macau, Macao 999078, China; sophia880728@126.com (F.S.); zhanglele@cdu.edu.cn (L.Z.); zegang8956@126.com (S.W.); yb67542@um.edu.mo (L.Y.); 2 School of Medicine, Chengdu University, Chengdu 610106, China

**Keywords:** deacetyl ganoderic acid F, LPS-induced inflammation, microglia cells, zebrafish, mice

## Abstract

Microglia mediated neuronal inflammation has been widely reported to be responsible for neurodegenerative disease. Deacetyl ganoderic acid F (DeGA F) is a triterpenoid isolated from *Ganoderma lucidum*, which is a famous edible and medicinal mushroom used for treatment of dizziness and insomnia in traditional medicine for a long time. In this study the inhibitory effects and mechanisms of DeGA F against lipopolysaccharide (LPS)-induced inflammation both in vitro and in vivo were investigated. On murine microglial cell line BV-2 cells, DeGA F treatment inhibited LPS-triggered NO production and iNOS expression and affected the secretion and mRNA levels of relative inflammatory cytokines. DeGA F inhibited LPS-induced activation of the NF-κB pathway, as evidenced by decreased phosphorylation of IKK and IκB and the nuclear translocation of P65. In vivo, DeGA F treatment effectively inhibited NO production in zebrafish embryos. Moreover, DeGA F suppressed the serum levels of pro-inflammatory cytokines, including TNF-α and IL-6 in LPS-stimulated mice model. DeGA F reduced inflammatory response by suppressing microglia and astrocytes activation and also suppressed LPS-induced NF-κB activation in mice brains. Taken together, DeGA F exhibited remarkable anti-inflammatory effects and promising therapeutic potential for neural inflammation associated diseases.

## 1. Introduction

Inflammation is a defense mechanism of the organism to various harmful stimuli, such as irritants, damaged cells, or pathogens [1]. Generally, inflammation is considered beneficial for self-healing and repairing owing to its function of eliminating the damaged tissues or necrotic cells from the original stimulation and the inflammatory reaction. However, an excessive or uncontrolled extent of inflammation is detrimental to an organism and may result in inflammatory disorders. Particularly, inflammation that occurs in the brain is widely recognized as a major cause of neurogenesis and neurodegenerative diseases [2,3]. Recent mechanistic studies and genetic evidence indicate that immune signal pathway dysregulation in brain; alternation in cytokine secretion, immune cell migration and proliferation, and abnormal phagocytosis and gliosis are common characteristics of neurodegenerative disorders [4]. Emerging evidence also supports the pivotal role of the immune system in disorder onset and progression [5,6,7]. In most neurodegenerative study, microglia, the resident innate immune cells in brain parenchyma, is regarded as the primary cellular mediator of the immune response. Microglia attract majority attentions since it possesses superior ability of cytokines modulating during inflammatory processes [8]. Under normal conditions, microglial cells display a low immunoreactivity and serve as immune surveillance and host defense. In response to pathogenic insult, microglial cells would be activated instantly and release various cytokines, including the pro-inflammatory cytokines (TNF-α, IL-6, IL-1β, etc.) and the anti-inflammatory cytokines (such as IL-10 and IL-4) [9,10]. The microglia mediated IL-6 overexpression was reported to be responsible for inhibition of neuron survival and decrease of new neuron production [11]. In contrast, the activated microglia enhanced IL-10 release was demonstrated to promote neuron survival and suppress continuous detrimental function of enlarged microglia cells [7].

*Ganoderma lucidum* (Fr.) Karst (Polyporaceae), which is called “Lingzhi” in Chinese, is a famous herbal medicine and edible mushroom in Asian countries for a long history [12]. As a nutritional supplement, Ganoderma tea is believed to be beneficial to health and is one of the most common form used in China by which the dry Ganoderma slices are soaked into hot water immediately. Nowadays, Ganoderma has been granted as a medicine food homology species by China Food and Drug Administration, and its extracts have even been processed into diversiform functional foods and nutraceuticals due to their health-care function and commercial value. Moreover, Ganoderma is clinically used to treat neurasthenia, debility from prolonged illness, anorexia, dizziness, and insomnia in traditional medicine. Modern pharmacological research has demonstrated that Ganoderma possesses outstanding biological effects, such as anti-inflammation and immunoregulation [13,14,15]. However, most of these studies focused on polysaccharides and triterpenes of Ganoderma; investigation on the monomeric compounds still remain inadequate [12,16]. 

Deacetyl ganoderic acid F (DeGA F) is a triterpenoid isolated from *Ganoderma lucidum*, which was firstly reported by Komoda at 1985 [17]. However, investigation on the bioactivity of DeGA F is very limited until now. In this study, the potential anti-inflammatory effects of DeGA F in lipopolysaccharide (LPS)-induced inflammation models were investigated both in vitro (BV-2 microglia cells) and in vivo (zebrafish and mice), and the mechanisms underlying the effects were further elucidated. 

## 2. Materials and Methods

### 2.1. Reagents

DeGA F was purchased from Chroma-Biotech (Chengdu, China). LPS, Escherichia coli 0111:B4, was acquired from Sigma (St. Louise, Missouri, MO, USA). Dulbecco’s modified Eagle’s medium (DMEM), phosphate buffered saline (PBS), fetal bovine serum (FBS), and penicillin-streptomycin (P/S) were obtained from Gibco (Gaithersburg, Maryland, MD, USA). GAPDH (#2118), iNOS (#13120), COX2 (#12282), p65 (#12282), IKKα (#11930), IKKβ (#8943), p-IKKα/β (Ser176/180) (#2697), p-IκBα (Ser32) (#2859), IκBα (#9242), and p-Akt (Ser473) (#4060) antibodies and relative secondary antibodies were acquired from Cell Signaling Technology (Boston, Massachusetts, MA, USA).

### 2.2. Cell Culture

BV-2 cells, an immortalized murine microglial cell line, were provided by the National Infrastructure of Cell Line Resource (Wuhan University, China), and were cultured in DMEM supplemented with 10% FBS (*v*/*v*) and 1% P/S (*v*/*v*) in a humidified chamber under 37 °C and 5% CO_2_ atmosphere. Cells were treated with relative concentrations of C11 for 1 h prior to LPS stimulation at 200 ng/mL.

### 2.3. Animal Maintenance and Administration

C57BL/6J mice (male, 20–25 g) aged 12–15 weeks were maintained in standard cages with 12-hour light/12-hour dark cycle under temperature of 21 ± 1 °C and humidity of 60 ± 5%, and allowed free access to water and food. The mice were then divided into 4 groups of 7 mice per group and orally gavage with vehicle, 5 and 10 mg/kg of DeGA F for 7 consecutive days. LPS (5 mg/kg body weight) was simultaneously injected intraperitoneally (except the blank group) at the 7th day after oral gavage 2 h later. All the mice were euthanized by rapidly cervical dislocation.

Wild-type zebrafish were maintained at standard boxes with a 14-hour light/10-hour dark cycle and constant temperature (28.5 ± 1 °C). Zebrafish embryos were placed individually into six-well plates of 15 embryos in each group containing 2 mL of medium. After 24 h post fertilization (hpf), embryos were pretreated with fresh medium or DeGA F for 1 h and then treated by LPS (5 µg/mL final concentration) for another 24 h in an incubator (28.5 ± 1 °C). The zebrafish were euthanized with an overdose of tricaine methane-sulfonate (MS-222) after experiments.

The protocol of this study was approved by the Animal Ethics Committee of University of Macau (UMARE-022-2017). All animal care and experimental procedures in this study were performed in strict accordance with the ethical guidelines for the care and use of laboratory animals in Institute of Chinese Medical Sciences, University of Macau.

### 2.4. Cell Viability

Cells were seeded in 96-well plates at a density of 1 × 10^5^ cells/mL. After treatment, Cell Counting Kit-8 reagents (CCK-8, Beyotime, Shanghai, China) were added into the cell culture medium (1:10, v:v) for 4 h incubation at a temperature of 37 °C, and then the absorbance at 450 nm was detected by a FlexStation III microplate reader (Molecular Devices, Sunnyvale, CA, USA).

### 2.5. Nitrite Production Determination

Cells were firstly treated with DeGA F for 1 h and then stimulated by 200 ng/mL of LPS for 24 h. Then, 100 µL of the cell culture medium were added in a new 96-well plate, mixed with 100 µL of Griess reagent (Beyotime, Shanghai, China) and then incubated for 15 min at 37 °C. The absorbance at 540 nm was detected.

Zebrafish embryos were administrated as described in the Section 2.3. The medium of each group was replaced with 1 µM of the DAF-FM DA solution (Invitrogen, Carlsbad, CA, USA) and incubate for 1 h, at 28.5 °C, in the dark. Then the embryos were rinsed off with fresh medium and anesthetized with tricaine MS-222 solution before observation.

### 2.6. Enzyme-Linked Immunosorbent Assay (ELISA)

Cells were treated as described above. After the cells were stimulated by LPS for 24 h, the supernatants of the cell culture medium were collected. The secretion levels of TNF-α and IL-6 were measured by using relative ELISA kits (Neobioscience, Shanghai, China) according to the manufacturer’s instructions. The collected supernatant of medium or serum were diluted with the sample dilution buffer at appropriate ratio. The absorbance at 490 nm was detected [18].

### 2.7. Western Blot Analysis

After treatment, the cells were lysed by RIPA lysis buffer supplemented with 1% cocktail (*v*/*v*) and 1% phenylmethanesulfonyl fluoride (*v*/*v*) for 20 min. For the mice samples, the brain tissues were homogenized to suspension in prior, and then they were lysed in RIPA lysis buffer for 20 min. Protein concentrations of the lysates were measured by BCA^TM^ Protein Assay Kit (Pierce, Rockford, Illinois, IL, USA). After heat denaturation, the proteins were immediately separated by SDS-PAGE and then transferred onto the polyvinylidene fluoride membranes, and further blocked with nonfat milk for 2 h at room temperature. The immunoreactions were carried out using GAPDH, iNOS, COX-2, p65, IKKα, IKKβ, p-IKKα/β, p-IκBα, IκBα and p-Akt antibodies, and then the membranes were probed with the corresponding secondary antibodies. Thereafter, the protein bands were visualized by the ECL Western Blotting Detection kit (GE Healthcare, Buckinghamshire, UK).

### 2.8. Quantitative Real-Time PCR (qPCR) Analysis

Total RNA of the cells was extracted by using the TRIzol reagent (Life Technologies, Shanghai, China). Mice brain tissues were homogenized, and the total RNA of the tissues was extracted by using the RNeasy Plus Mini Kit (Qiagen, Hilden, Germany). Concentrations of the RNA samples were determined and then the samples were reverse-transcribed using a PrimeScript RT Reagent Kit (Takara, Shiga, Japan). Thereafter, qPCR analysis was carried out quantitatively on a ViiA 7 qPCR System (Life Technologies, Carlsbad, California, CA, USA) in accordance with SYBR assay. The primer sequences are listed in Table 1.

### 2.9. Immunocytochemistry

The mice brain sections were separated and then fixed by 4% paraformaldehyde (Sigma-Aldrich, St Louis, Missouri, MO, USA). After three-times washing with PBS, the tissues were then permeabilized with the blocking buffer (0.3% Triton X-100, 10% goat serum in PBS). Then the tissues were incubated with antibodies against Iba-1 (#NCNP24, Wako, Osaka, Japan) or GFAP (#ab4674, Abcam, Cambridge, Massachusetts, MA, USA) overnight at 4 °C and further incubated with relative secondary antibodies for another 1 h, at room temperature. Thereafter, the brain slices were stained with 5 μg/mL of DAPI (Sigma-Aldrich, USA) for 15 min. Images were photographed by using a Leica TCS SP8 confocal laser scanning microscope (Solms, Germany).

### 2.10. Statistical Analysis

Data were presented as mean ± standard deviation (SD). The statistical significance was analyzed by one-way analysis of variance using the GraphPad Prism program 6.0 (San Diego, California, CA, USA). */^#^ *p* < 0.05 and **/^##^ *p* < 0.01 were considered statistically significant.

## 3. Results

### 3.1. DeGA F Inhibited NO Production and iNOS Expression in LPS-Stimulated BV-2 Cells

**Figure 1:**
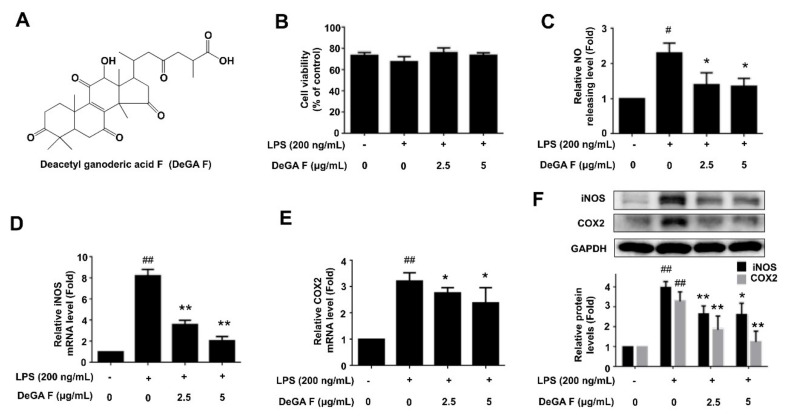
Deacetyl ganoderic acid F (DeGA F) inhibited Nitric oxide (NO) production and iNOS expression in LPS-stimulated BV-2 cells. (**A**) Chemical structure of DeGA F. (**B**) Cells were pretreated with DeGA F for 1 h, and then exposed to LPS for another 24 h. Cell viability was detected using CCK-8 assay. (**C**) NO releasing levels in the cell culture medium were detected by Griess assay. (**D**,**E**) The mRNA levels of iNOS and COX-2 were measured by qPCR analysis. (**F**) Protein levels of iNOS and COX-2 were detected by Western blot analysis. LPS, lipopolysaccharide. – and + represented the absence or presence of LPS (200 ng/mL), respectively. ^#^ *p* < 0.05 and ^##^ *p* < 0.01 compared with blank group (*n* = 3). * *p* < 0.05 and ** *p* < 0.01 compared with the LPS group (*n* = 3).

The chemical structure of DeGA F is illustrated in Figure 1A. The effect of DeGA F on BV-2 cell viability was evaluated by using CCK-8 assay. BV-2 cells were pretreated with DeGA F for 1 h and then stimulated by LPS for another 24 h. The results indicated that DeGA F was nontoxic to the BV-2 cells up to 48 h (Figure 1B), and morphological changes in the cells were rarely observed in the microscopic analysis (data not shown). Thus, concentrations of 2.5 and 5 µg/mL that didn’t induce cell death were selected for further study.

Nitric oxide (NO) is a major mediator of inflammatory response. Excessive production of NO is a hallmark of LPS-triggered inflammatory response [19,20]. To determine the effects of DeGA F on NO production of LPS-stimulated BV-2 cells, nitrite level, the stable NO metabolite in the cell medium was tested by using the Griess regents. As shown in Figure 1C, NO level increased after LPS challenge, while DeGA F treatment could significantly inhibit the increase of NO production caused by LPS in BV-2 cells. Thereafter, the expression of iNOS and COX-2, the pro-inflammatory mediators for NO generation, were investigated to explain the inhibitory effect of DeGA F on NO overproduction. As expected, LPS treatment resulted in about 8.2-fold and 3.2-fold increase in mRNA levels of iNOS and COX-2. Pretreatment with 2.5 and 5 µg/mL of DeGA F markedly decreased mRNA levels of iNOS to about 3.6-fold and 2.1-fold, and decreased mRNA levels of COX-2 to about 2.7-fold and 2.3-fold, respectively (Figure 1D,E). Moreover, the results of Western blot analysis also confirmed that DeGA F pretreatment inhibited the upregulation of iNOS and COX-2 protein levels induced by LPS stimulation (Figure 1F). These results suggested that DeGA F inhibited the accumulation of NO by regulating the iNOS and COX-2 expression, and it might be a potential inhibitor of microglial activation.

### 3.2. DeGA F Inhibited LPS-Induced Inflammatory Cytokine Release in BV-2 Cells

**Figure 2:**
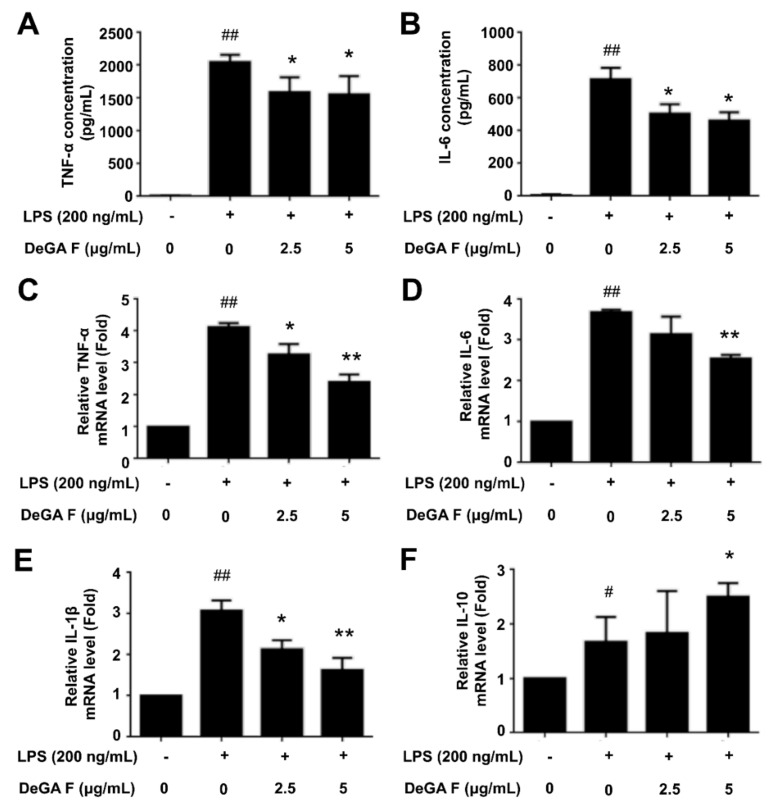
DeGA F affected the secretion and mRNA levels of the inflammatory cytokines in LPS-stimulated BV-2 cells. Cells were pretreated with DeGA F for 1 h, and then exposed to LPS for another 24 h. (**A**,**B**) Secretion levels of TNF-α and IL-6 in the cell culture medium were determined by relative ELISA kit. The mRNA expression levels of TNF-α (**C**), IL-6 (**D**), IL-1β (**E**), and IL-10 (**F**) were measured by qPCR analysis. – and + represented the absence or presence of LPS (200 ng/mL), respectively. ^#^ *p* < 0.05 and ^##^ *p* < 0.01 compared with blank group. * *p* < 0.05 and ** *p* < 0.01 compared with the LPS group (*n* = 3).

In addition to NO overproduction, a series of inflammatory cytokines are also involved in inflammatory process once the microglia is activated by LPS. Herein, we firstly determined the secretion levels of TNF-α and IL-6 in LPS-stimulated BV-2 cell culture medium in the absence or presence of DeGA F by ELISA assay. As illustrated in Figure 2A,B, LPS treatment increased the secretion of TNF-α and IL-6, whereas pretreatment with 2.5 and 5 μg/mL of DeGA F attenuated the trends, indicating that DeGA F could inhibit pro-inflammatory cytokines secretion in activated microglia. To verify this result, the mRNA levels of the relative cytokines were further detected. qPCR analysis showed that DeGA F effectively suppressed LPS-induced upregulation in the mRNA levels of TNF-α (Figure 2C), IL-6 (Figure 2D), and IL-1β (Figure 2E). On the other hand, the mRNA level of the anti-inflammatory cytokine member IL-10 increased upon LPS stimulation, while DeGA F pretreatment further promoted this trend (Figure 2F). Therefore, DeGA F suppressed LPS-induced inflammatory reaction not only by downregulating the pro-inflammatory cytokines, but also via upregulating the anti-inflammatory cytokines.

### 3.3. DeGA F Suppressed LPS-Triggered Inflammatory Response via NF-κB Pathway

**Figure 3:**
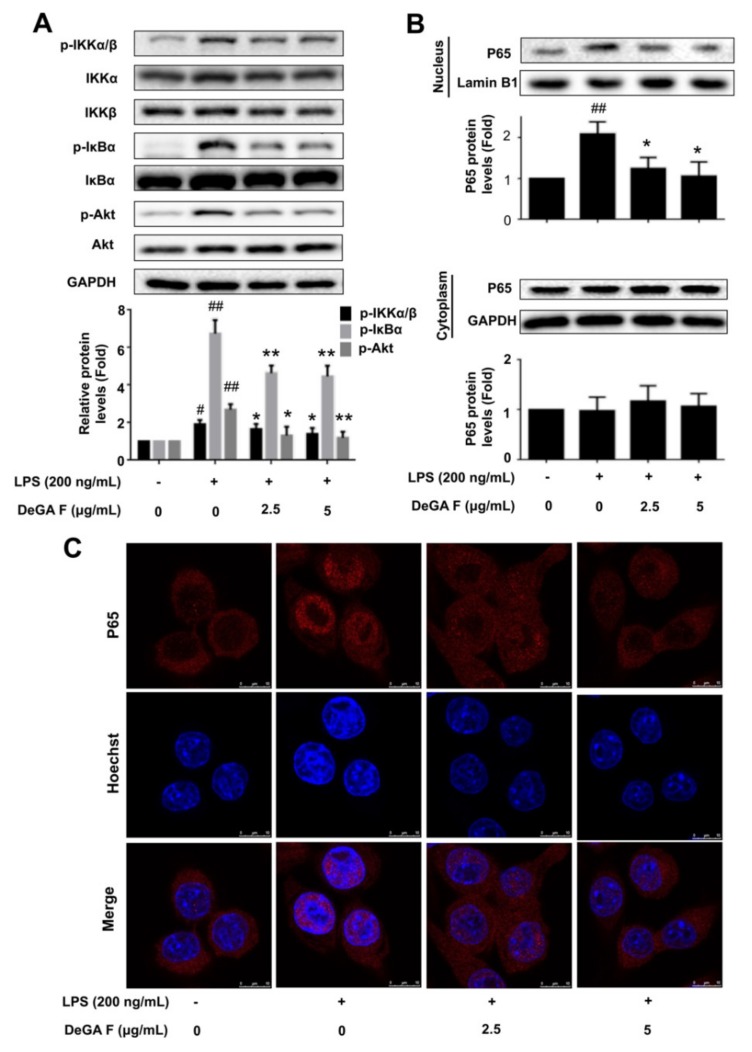
DeGA F suppressed LPS-triggered activation of NF-κB pathway in BV-2 cells. Cells were pretreated with DeGA F for 1 h, and then exposed to LPS for another 3 h. (**A**) Expression of relative proteins were determined by Western blot analysis. (**B**) Nuclear and cytoplasmic proteins were extracted and expression levels of P65 were detected by Western blot. (**C**) Nuclear translocation of P65 was evaluated by immunofluorescence analysis. Cells were stained with anti-P65 antibody (red) and Hoechst (blue). – and + represented the absence or presence of LPS (200 ng/mL), respectively. ^#^ *p* < 0.05 and ^##^ *p* < 0.01 compared with blank group. * *p* < 0.05 and ** *p* < 0.01 compared with the LPS group (*n* = 3).

Subsequently, whether DeGA F could affect the LPS-induced activation of the NF-κB signaling pathway was investigated. As shown in Figure 3A, subjecting with LPS could promote phosphorylation of Akt, IKKα/β and IκBα in microglia BV-2 cells. Pretreatment with DeGA F obviously inhibited p-Akt, p-IKKα/β and p-IκBα expression compared with the LPS stimulation group. To detect the nuclear translocation of P65, the nuclear and cytoplasmic protein levels of the BV-2 cells were analyzed using Western blot. As shown in Figure 3B, the nuclear levels of P65 increased in response to LPS stimulation, while DeGA F pretreatment obviously inhibited P65 nuclear translocations. Moreover, immunofluorescence analysis further confirmed the inhibitory effect of DeGA F against LPS-induced nuclear translocation of P65 (Figure 3C). These results indicated DeGA F to be a potential inhibitor against LPS-induced NF-κB activation.

### 3.4. DeGA F Suppressed NO Production in LPS-Stimulated Zebrafish Model

**Figure 4:**
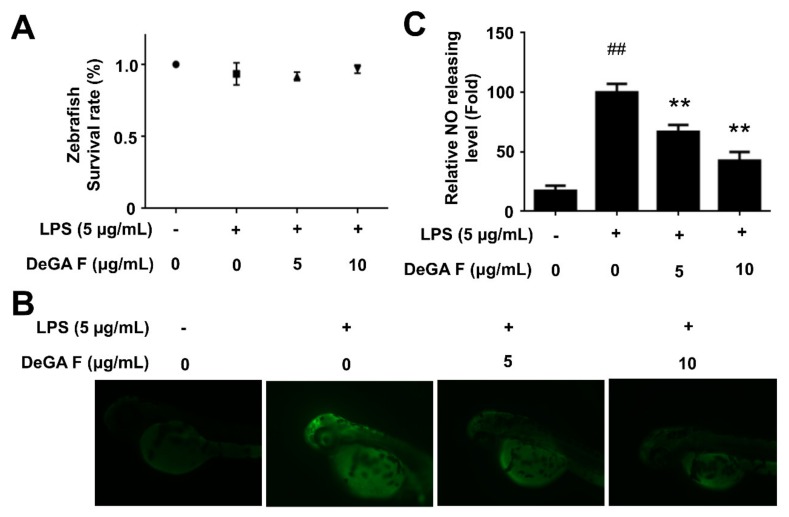
DeGA F suppressed NO production in LPS-triggered zebrafish model. Zebrafish embryos were pretreated with DeGA F for 1 h, and then stimulated with LPS for another 24 h. (**A**) Toxicity of DeGA F (≤100 µg/mL) on zebrafish model. (**B**) The NO level was determined by staining fluorescent probe DAF-FM DA for 1 h. (**C**) The results were calculated by ImageJ software. ^#^ *p* < 0.05 and ^##^ *p* < 0.01 compared with blank group (*n* = 15). – and + represented the absence or presence of LPS (200 µg/mL), respectively. * *p* < 0.05 and ** *p* < 0.01 compared with the LPS group (*n* = 15).

The zebrafish is a recognized model for histological and physiological studies, especially in discovery of promising compounds with anti-inflammation activity since the zebrafish has been documented to possess similar innate and acquired immune systems closely like Mammals [21,22,23,24]. A zebrafish embryo is characterized by optical transparency, which allows noninvasive and dynamic imaging of the inflammatory response in vivo [19]. Moreover, it facilitates analysis of the immune response since the innate immune system of a zebrafish embryo only exists in early weeks postfertilization. Taking advantages of the above features, the fluorescent probe DAF-FM was used to determine NO level in LPS-stimulated zebrafish model. Toxicity of DeGA F was assessed prior. The results indicated that DeGA F was nontoxic to zebrafish embryo in 3 dpf, even at a concentration of 10 µg/mL (Figure 4A). Embryo insulted by LPS exhibited organ failure, such as lethargy, raised heart beating and edema. Nevertheless, exposed to DeGA F significantly decreased organ toxicities in certain extant (data not shown). As shown in Figure 4B, LPS treatment obviously elevated NO level compared with that of the control group. Similar to the results in microglia cells, DeGA F attenuated the increasing NO production induced by LPS. Particularly, 10 µg/mL of DeGA F inhibited NO production in zebrafish embryo by over 50% (Figure 4C). This result was well correlated with the in vitro data in microglia cell and supported the anti-inflammatory effect of DeGA F in vivo.

### 3.5. DeGA F Attenuated LPS-Triggered Inflammatory Response in Mice

**Figure 5:**
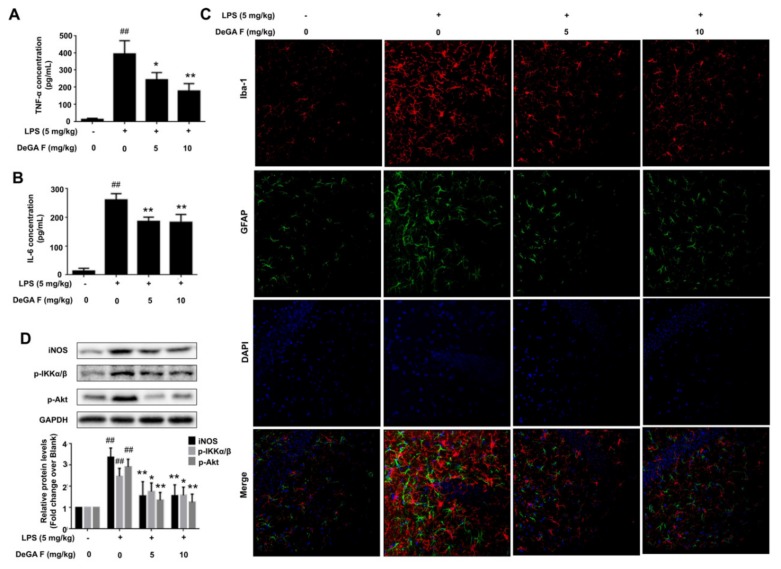
DeGA F attenuated LPS-triggered inflammatory response in mice. Mice were daily administrated with DeGA F for five days, and then subjected to LPS (i.p. 5 mg/kg) 2 h later, on the fifth day. The serum levels of TNF-α (**A**) and IL-6 (**B**) were determined by ELISA. (**C**) Immunofluorescence analysis of mice brain tissues (20 µm). The tissues were stained with rabbit anti-Iba-1 antibody (red) and mouse anti-GFAP antibody (green). The nuclei were stained with DAPI (blue). (**D**) Expression of inflammation related proteins in mice brain were detected by Western blot analysis. ^#^ *p* < 0.05 and ^##^ *p* < 0.01 compared with blank group (*n* = 7). – and + represented the absence or presence of LPS (5 mg/kg), respectively. * *p* < 0.05 and ** *p* < 0.01 compared with the LPS group (*n* = 7).

The anti-inflammatory effects of DeGA F were demonstrated in BV-2 cells and zebrafish. Next, whether DeGA F could suppress LPS-induced inflammation in mice model was assessed. Herein, mice were daily administrated with different doses of DeGA F for five days, and then subjected to LPS (i.p. 5 mg/kg) 2 h later, on the fifth day. After LPS challenge, mice in LPS group appeared sickness responses including tearing, lethargy, twitch symptoms and decreased locomotor activity. However, these behavior responses like twitch and over secretion of tears were improved in mice treated with DeGA F. We next detected the release of the key pro-inflammatory cytokines in mice serum. As illustrated in Figure 5A,B, the serum levels of TNF-α and IL-6 showed pronounced increase after LPS exposure, and was partially suppressed by DeGA F treatment. 

Iba-1 and GFAP represent specific biomarkers for microglia and astrocytes respectively [25,26]. To visualize the inhibitory effect of DeGA F on LPS invoked mice model, the status of microglia and astrocytes in mouse brain were then investigated by using double-staining of Iba-1 and GFAP. As shown in Figure 5C, the expression of Iba-1 was upregulated significantly after LPS treatment. The population of Iba-1 positive cells in the model group increased obviously, and morphology of the cells altered to reactive status which was characterized by larger and thicker cell morphology and stout branches compared with that in the control group. DeGA F treatment attenuated LPS-induced microglia activation as the number of Iba1-positive cells decreased obviously compared with model group, and most of the cells recovered to normal morphology. Similarly, GFAP, a structural protein of astrocytes and the marker proportional to the level of reactivity [27], was also upregulated in the LPS group (Figure 5C). The morphological features of LPS challenged astrocytes include hypertrophy of cell soma and processes, overlapping domains. With DeGA F treatment, the morphology of individual astrocytes was well maintained, almost non-overlapping domains and less cellular hypertrophy were observed. These results indicated that DeGA F reduced inflammatory response by suppressing microglia and astrocytes activation.

Beside the evaluation of morphology change and immunofluorescence analysis of Iba-1 and GFAP, expression of inflammation related proteins in mice brain were detected using Western blot. Similarly, we found that DeGA F treatment dramatically suppressed LPS-induced upregulation of iNOS, p-Akt, and p-IKKα/β in mice (Figure 5D). Briefly, these results further confirmed the data acquired in BV-2 cells. However, the protein level of IκBα could not be detected by Western blot in the tissue samples, which might be partially due to that the brain tissue consists of various cells (such as neurons), and the microglia is not the dominate part. In addition, inflammatory response is a dynamic time-related procedure. Different factors may only participate in typical sections during the procedure. Herein, we just investigated the changes at the time point of 2 h, which may be unable to illustrate all the alternations during the inflammation process.

## 4. Discussion

Neurodegenerative diseases, which are characterized by cognitive decline, severe motor disability, and dementia, include Parkinson’s disease (PD), Alzheimer’s disease (AD), Huntington’s disease (HD), and amyotrophic lateral sclerosis (ALS). There is general agreement on considering the inflammatory response by innate immune system of the CNS is highly involved in development of neurodegenerative diseases [28]. Innate immune system is the first line to defense insult, but also is crucial in tissue repair and clearance of waste products, such as apoptotic cells and tissue debris [29]. Although the innate immune system takes a quite complex role, the inflammatory response in the brain is mainly detrimental, especially by activated glia (the microglia and astrocytes) mediated abnormal secretion of proinflammatory and anti-inflammatory cytokines [29,30]. The microglia and astrocytes are two key players that represent the innate immune system in the CNS; therefore, the activated status of these two cells were usually observed and illustrated to evaluate the degree of inflammation in the brain [5,10,31].

NO is a short-lived radical generated by iNOS, and its overexpression is wildly demonstrated as being a cytotoxic indicator of inflammatory response [32,33]. Therefore, iNOS generated NO is usually detected to assess the inflammatory process. Beside NO, many other inflammatory mediators are secreted during the inflammatory process, for example, the prostaglandins. The COX-2 isozyme catalyzes the inducible production of prostaglandins, which represents a significant step in inflammatory response. Therefore, COX-2 is an important element in inflammation, and inhibition of its expression by DeGA F might further induce the suppression of prostaglandins production to a certain extent. Pro-inflammatory cytokines participate in defense mechanisms of the immune cells, but their excessive release may lead to immunopathological disease [4,7]. TNF-α, IL-6 and IL-1β are representative members of pro-inflammatory cytokines, which are secreted in excess at early stages, generally, and may further amplify inflammation [11]. In contrast, IL-10, a major member of anti-inflammatory cytokines, can reduce the triggered reaction and promote self-healing [2,34]. In this study, DeGA F showed dual regulatory effects that decreasing pro-inflammatory cytokines production, while increasing anti-inflammatory cytokines generation. 

To date, a number of signaling pathways have been documented to participate in the LPS-triggered inflammatory response. The NF-κB family of transcription factors is generally believed to play a vital role in regulating the expression of genes that are involved in mammalian immune systems [22,35,36]. Normally, NF-κB dimers stay in cytosol as an inactive form by interacting with IκBα family protein. Once the inhibitory IκBα protein dissociates, NF-κB will be activated, and the liberated NF-κB dimers will translocate into the nucleus [37]. P65 is one of the most predominant proteins in NF-κB family which forms homodimers and heterodimers that bind to DNA sequences to regulate target gene expression, and the P65 heterodimer is a vital member for transcription regulation of NF-κB in the nervous system. In canonical NF-κB signaling pathway, stimuli such as LPS and TNF-α may activate IκBα kinase (IKK), and then the activated IKK would further phosphorylate IκBα and finally result in nuclear translocation of the liberated NF-κB dimer [38]. Previously, numerous genes have been identified to be the downstream targets of p65, and some of them were reported to be associated with the secretion of inflammatory cytokines. In particular, TNF-α, IL-1β, and IL-6 were also demonstrated to be target genes of p65. Nuclear translation of p65 might promote the expression and secretion of these cytokines. Furthermore, Akt has been reported to participate in cell defense and immune regulation as an upstream regulator of NF-κB [39,40]. Activation of Akt results to inflammation by stimulating IKK phosphorylation in the NF-κB pathway [41]. To clarify the precise anti-inflammatory mechanism of DeGA F, the relationship between the inhibitory effects of DeGA F on Akt and the NF-κB pathway need to be investigated.

## 5. Conclusions

In this work, we demonstrated that DeGA F inhibited LPS-stimulated inflammatory response in BV-2 cells, as evidenced by the suppression of NO production and pro-inflammatory cytokines secretion. Modulation of the NF-κB pathway may be a dominate mechanism underlying the anti-inflammatory effects of DeGA F. In addition, DeGA F suppressed NO production in LPS-stimulated zebrafish model and inhibited the serum levels of pro-inflammatory cytokines TNF-α and IL-6 in LPS stimulated mice model. DeGA F reduced inflammatory response by suppressing microglia and astrocytes activation and also suppressed LPS-induced NF-κB activation in mice brain. Totally, this study provided evidence for the therapeutic potential of DeGA F in neural inflammation associated diseases.

## Figures and Tables

**Table 1:** Primers of the investigated genes in qPCR analysis.

Gene		Primer Sequences
GAPDH	F	GGTGAAGGTCGGTGTGAACG
	R	CTCGCTCCTGGAAGATGGTG
iNOS	F	GGCTGTCAGAGCCTCGTGGCTTTGG
	R	CCCTTCCGAAGTTTCTGGCAGCAGC
COX2	F	TTGAAGACCAGGAGTACAGC
	R	GGTACAGTTCCATGACATCG
TNF-α	F	GGCAGGTCTACTTTGGAGTCATTGC
	R	ACATTCGAGGCTCCAGTGAATTCGG
IL-6	F	CCACTTCACAAGTCGGAGGCTT
	R	CCAGCTTATCTGTTAGGAGA
IL-1β	F	GGCAACTGTTCCTGAACTCAACTG
	R	CCATTGAGGTGGAGAGCTTTCAGC
IL-10	F	ATAACTGCACCCACTTCCCA
	R	GGGCATCACTTCTACCAGGT
